# Career Motivations, Postgraduate Specialties Interest, and Preferred Careers Upon Graduation Among Dental Students in the Northern Region of Saudi Arabia—A Cross-Sectional Survey Study

**DOI:** 10.1155/sci5/8351405

**Published:** 2025-10-09

**Authors:** Rakhi Issrani, Amal Alrayes, Sunitha Siddanna, Muna M. AlAli, Muhammad Nadeem Baig, Ahmed Hamoud L. Alsharari, Kiran Kumar Ganji, Namdeo Prabhu

**Affiliations:** ^1^Department of Preventive Dentistry, College of Dentistry, Jouf University, Sakaka, Saudi Arabia; ^2^Department of Public Health Dentistry, JSS Dental College and Hospital, JSS Academy of Higher Education and Research, Mysore, India; ^3^Department of Prosthodontics, Ministry of Health, Riyadh, Saudi Arabia; ^4^Department of Research Analytics, Saveetha Dental College and Hospitals, Saveetha Institute of Medical and Technical Sciences, Saveetha University, Chennai, India; ^5^Department of Oral & Maxillofacial Surgery and Diagnostic Sciences, College of Dentistry, Jouf University, Sakaka, Saudi Arabia

**Keywords:** dental education, dental graduates, dental specialties

## Abstract

**Background:**

It is essential to identify the factors that influence a student's choice of dental career, interest in postgraduate specialties, and preferred careers upon graduation when planning the dental workforce agenda in the country.

**Objectives:**

We examined the factors that influenced undergraduate (UG) students of Jouf University, Saudi Arabia, to choose dentistry as a profession. We also assessed students' preferences for dental specialties following graduation and immediate career plans upon graduation and whether they differed by demographics.

**Methodology:**

This was a cross-sectional survey study that was conducted from November 15, 2021, to January 15, 2022. A twelve-item questionnaire was hand-delivered to the third-, fourth-, and fifth-year UG students and interns of the College of Dentistry, Jouf University. The research questions focused on demographic information, reasons for choosing dentistry as a profession, preferences for postgraduate studies, and preferred career upon graduation. Mann–Whitney U and chi-square tests were performed.

**Results:**

A total of 144 students responded to the survey. The samples consisted of 84 (58.3%) male and 60 (41.7%) female students. The reasons for choosing dentistry as a career were mostly related to personal and vocational factors. Significant differences were noticed between male and female respondents for most of the reasons for choosing dentistry as a career (*p* ≤ 0.05). The female respondents mostly preferred *restorative and esthetic dentistry* as a specialty of choice, whereas the male participants preferred *oral and maxillofacial surgery*. The most preferred career among male and female respondents was “civilian dentist in the public sector.” There was no significant difference among genders with respect to the most preferred specialty and immediate career plans upon graduation (*p* > 0.05).

**Conclusion:**

Personal and vocational factors influenced the career preferences, with esthetic dentistry as the most preferred specialty for the graduates to choose. These data might aid the policy makers of the university for student career guidance.

## 1. Introduction

Choosing a career is one of the most important decisions one has to make, as it lays down the path of one's life [1]. Dentistry is increasingly chosen as a preferred career by the youth today, primarily due to financial security and perceived work-life balance [2]. Focusing on students' enthusiasm for selecting dentistry is crucial to understanding their motivations [3, 4]. Previous studies have reported that the majority of the undergraduate (UG) dental students preferred to pursue postgraduate studies soon after graduation [5–7]. Postgraduate professionals are essential in meeting the specific dental health needs of society, particularly where general dentistry is insufficient. Moreover, postgraduate education plays a vital role in teaching and research in dentistry [8].

The establishment of dental schools in Saudi Arabia dates back to 1987, when the government took strides to conduit the gap between dentists and the general populace, which could be attributed to the increase in population and the need for oral health professionals to serve the nation [9]. In Saudi Arabia, it is mandatory for a student to complete a 6-year dental program that includes a 1-year compulsory internship from an accredited educational setup to become a dental graduate [10]. In recent years, an increase in the number of dental schools within the kingdom has in turn been associated with an increase in the number of dental graduates [11]. This expansion of dental colleges would probably have its implications on the job market and perhaps the quality of dental practice in Saudi Arabia [11]. At such a critical moment, decision-makers and policy planners for postgraduate studies need to address this issue. Furthermore, effective management of the future dental workforce in Saudi Arabia would also require a thorough understanding of students' motivation for choosing dentistry as a profession. Moreover, as part of comprehensive planning for dental education in Saudi Arabia, there is a need to assess the intent of Saudi dental students to advance in their dental education and pursue postgraduation [10–13].

While various studies have explored motivations for career selection in different regions [10, 14], limited data exist from the northern region of Saudi Arabia. Hence, this study aimed to assess the factors influencing UG dental students at Jouf University in choosing dentistry, their postgraduate specialty preferences, and their career plans after graduation, and to examine the influence of demographic factors.

## 2. Materials and Methods

### 2.1. Study Design

A cross-sectional survey study was planned. The study was conducted as per the guidelines of Strengthening the Reporting of Observational Studies in Epidemiology (STROBE) [15].

### 2.2. Study Duration

This cross-sectional survey study was conducted from November 15, 2021, to January 15, 2022.

### 2.3. Sample Size

Using “Estimating Single Proportion with Finite population correction,” sample size estimation was done. A sample size of 130 was found to be adequate considering a 40% prevalence of career satisfaction (as determined by the pilot study that was carried out with 10 randomly chosen participants from the study site), an absolute precision of 5%, a 95% confidence interval, and a target population size of 200.

### 2.4. Conceptual Framework

The conceptual framework of this study is grounded in vocational choice theories, especially Holland's Theory of Career Choice [16] and Gottfredson's Theory of Circumscription and Compromise [17]. These theories emphasize the interaction of personal interests, societal expectations, and perceived competencies in career decision-making. This framework supports the categorization of our questionnaire into five conceptual domains: financial, professional, vocational, social, and personal.

### 2.5. Research Questions

A twelve-item questionnaire was prepared based on knowledge from previous research [10, 14]. The following three sections were included in the questionnaire:

1. Section I consisted of background information (age, gender, and academic year)—Questions 1–3;
2. Section II comprised 12 reasons related to students' decision to study dentistry that were measured on a 5-point Likert scale anchored by *strongly agree* (1) to *strongly disagree* (5). To facilitate analysis, the listed reasons were categorized into financial, professional, vocational, social, and personal reasons—Questions 4–8; and
3. Section III comprised students' preferences for dental specialty after graduation and immediate career plans upon graduation measured. The responses were measured on a 5-point Likert scale anchored by *first choice* (1) to *never* (5)—Questions 9–12. The students were asked to indicate their best preference for postgraduate specialties among the list of 13 dental specialties, along with general dentistry as an option. Regarding preferred career plans upon graduation, the students were given 7 choices along with “other careers” as an option.

### 2.6. Procedure

Two senior faculty members with expertise in survey research performed the face and content validation of the questionnaire. To determine how well respondents could comprehend and reply to the offered questionnaire, pilot research was carried out with 10 randomly chosen participants from the study site. Although the pilot sample was limited, it was drawn from a homogeneous group of student representatives of the study population. Reliability analysis yielded Cronbach's alpha coefficients ranging from 0.949 to 0.954 across different sections, indicating excellent internal consistency. According to Johanson and Brooks, small pilot samples may still produce valid reliability estimates when the internal consistency is high [18].

The questionnaire items were mapped to five theoretical domains derived from vocational choice theory: financial (Q4), professional (Q5), vocational (Q6), social (Q7), and personal (Q8) reasons. This mapping was used to align questionnaire content with the conceptual constructs of the study.

The third-, fourth-, and fifth-year UG students and interns were the target population because we assumed that they had sufficient exposure to dentistry to for a sound opinion about their choices for the present and future of their dental careers.

Systematic random sampling was used for participant selection. The first subject was selected through lottery method and then every 2nd subject (based on the roll numbers of students) was included in the study till the target sample size was achieved. Principal investigator hand-delivered the questionnaire to the selected UG students and interns of College of Dentistry, Jouf University, Saudi Arabia and collected them the next day. A written informed consent was obtained from all the participants who were assured that the results of the survey would be only presented or published as an aggregate data maintaining the confidentiality of personal information.

### 2.7. Statistical Analysis

Data was analyzed using Statistical Package for Social Sciences Version 20.0 (IBM Corp., Armonk, NY, USA). Descriptive statistics presented demographic data of respondents, and a frequency table was generated to illustrate the response of students to questionnaire items. The nonparametric Mann–Whitney U-test was used to identify any differences among students' groups in their reasons for choosing dentistry as a career. Comparisons of inclination toward pursuing postgraduation or general dentistry and immediate career plans were evaluated using the chi-square test. The level of statistical significance was set at *p* ≤ 0.05.

## 3. Results

A total of 144 students participated in this study. The study comprised 84 (58.3%) male and 60 (41.7%) female students, with the mean age of participants being 23.2 ± 2.0 years. Since there are currently only three batches of female students in this institute as opposed to five batches of male students, the percentage of female students who responded to the survey was lower than that of male students. As a result, gender differences, if any, may need to be interpreted with caution. Among 144 respondents, 47 (32.6%) were 3^rd-^year students, 25 (17.4%) were 4^th^-year students, 47 (32.6%) were 5^th^-year students, and 25 (17.4%) were interns (Table 1).

### 3.1. Reasons for Choosing Dentistry as a Profession

The reasons for choosing dentistry as a career among the surveyed dental students were mostly related to personal and vocational factors. The top three items for choosing dentistry as a career were, firstly, “I like to improve the esthetics of people,” “I have good manual dexterity and skills to practice dentistry,” and “my school GPA qualified me to choose dentistry as a career.” Respondents strongly agreed that these items were highly influential on their decision to pursue dentistry as a profession.

The statistical comparison between genders indicated that the professional (for the statement “I can be my boss”) and social reasons had a significantly higher impact on male students in their choice of dentistry as a career (*p* < 0.05). On the other hand, the economic, professional (for the statement “I can practice dentistry independently after my graduation with no need to be a specialist” and “Dentistry offers more regular work hours that other medical courses”), and personal reasons were significantly more influential on female students' decision to study dentistry (*p* < 0.05) (Table 2).

### 3.2. Preferences for Choosing Specialty Dentistry or General Dentistry

Analysis of students' responses regarding the preferences for choosing a specialty for postgraduate studies or continuing as a general practitioner revealed that the vast majority of participants (98.6%) expressed a desire to follow a postgraduate study. Among the different dental specialties, *restorative and esthetic dentistry* was the most preferred specialty (*n* = 46; 31.9%), followed by *oral and maxillofacial surgery* (*n* = 40; 27.8%), *endodontics* (*n* = 15; 10.4%), and *orthodontics* (*n* = 14; 9.7%).

The most preferred specialty among female respondents was *restorative and esthetic dentistry* (*n* = 22; 46.8%), followed by *oral and maxillofacial surgery* (*n* = 13; 44.8%) and *orthodontics* (*n* = 10; 34.4%). The most preferred specialty among male respondents was *oral and maxillofacial surgery* (*n* = 27; 45.0%), followed by *restorative and esthetic dentistry* (*n* = 24; 36.9%) and *endodontics* (*n* = 12; 22.6%). There was no significant difference among genders with respect to the most preferred specialty (*p* > 0.05).

**Figure 1:**
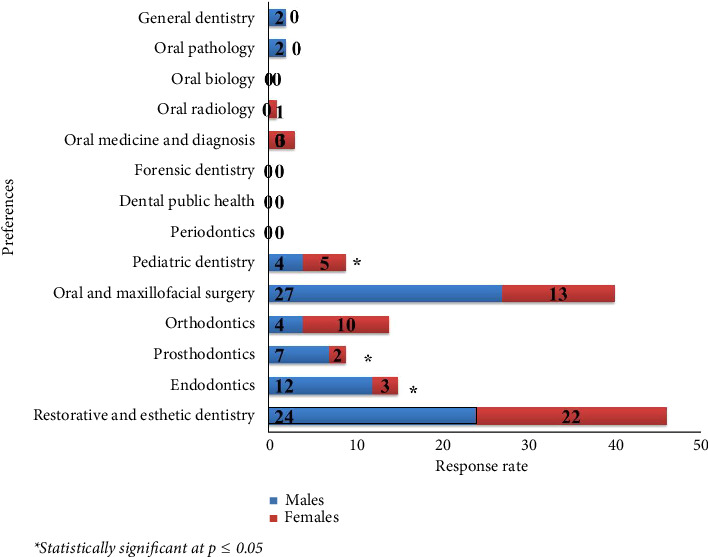
Gender-wise preferences of dental specialties/general dentistry.

The distribution of specialties/general dentistry preferences based on gender is given in Figure 1.

### 3.3. Preferences for Career Plans Upon Graduation

The three most preferred careers were “civilian dentist in public sector” (*n* = 83; 63.4%), followed by working as a “dentist in military sector” (*n* = 21; 25.6%), and “academic services dentist” (*n* = 16; 15.8%).

The most preferred career among female respondents was “civilian dentist in public sector” (*n* = 41; 73.2%), followed by “academic services dentist” (*n* = 6; 15.8%), “management of dental business” (*n* = 4; 19.0%), and “researcher” (*n* = 4; 30.7%). The most preferred specialty among male respondents was “civilian dentist in public sector” (*n* = 42; 56.0%), followed by “dentist in military sector” (*n* = 19; 31.7%) and “academic services dentist” (*n* = 10; 15.9%). There was no significant difference among genders with respect to the most preferred careers upon graduation (*p* > 0.05).

The distribution of the respondents according to the most preferred career upon graduation is shown in Table 3.

## 4. Discussion

Several factors have been included in the literature concerning the choice of a career in dentistry [10]. Factors like social standing [14, 19]; high professional status [19]; higher social status and income [19–23]; ability to be self-employed [20, 21, 24]; influence of family members in the dental profession [10]; artistic nature of the career [6, 20, 21, 24, 25]; and helping people and general interest in dentistry [6, 20, 21, 24] have been reported as the most important factors influencing their choice of career. Holland's theory reinforces the role of personality fit in career choices, and Gottfredson emphasized social influences and constraints, both relevant in interpreting our participants' responses [16, 17]. The demographic characteristics of the population are also an important determinant that influences the motivations and career expectations among dental students [10]. Shaker and Babgi had reported that approximately half of the dentists graduating from the two oldest dental schools in Saudi Arabia were females [26]. Also, Al-Hallak et al. reported that the proportion of female students exceeded the proportion of male students in their study [14]. The majority of respondents in the present study were males as compared to the female participants. This could be attributed to the lesser number of female students attending the dental college, as only three batches of female students are currently present in this institute as compared to the five batches of male students. In this study, on questioning for the reasons to choose dentistry as a career, the majority of the participants bestowed the reason related to personal and vocational factors. The statistical comparison among genders indicated that the professional and social reasons had a significantly higher impact on male students in their choice of dentistry as a career. On the other hand, the economic, professional, and personal reasons were significantly more influential on female students' decision to study dentistry. This is inconsistent with the findings of Al-Hallak et al., in which the personal and economic factors had a greater impact on male students' decision to study dentistry, whereas female students were more influenced by professional and vocational factors [14]. Further work to explain this difference among genders is recommended.

This study also showed that most of the participants were highly motivated to pursue postgraduate studies and to continue the specializing journey. This is consistent with the study done by Halawany et al. and Al-Hallak et al., wherein the majority of the studied participants expressed their wish to pursue postgraduate studies [10, 14]. Furthermore, one institutional study conducted in Saudi Arabia reported that among 532 male (1982–2004) and 545 female graduates (1984–2006), 77.0% and 54.0%, respectively, successfully completed their postgraduate dental education [27, 28]. This means that more training opportunities and postgraduate programs should be planned to accommodate the speculated increasing demand for higher education in Saudi Arabia.

The specialty preferences of Saudi dental students seem to have changed since the last decade [10]. This study's findings regarding students' motivations and career preferences are supported by international studies. For instance, Shetty et al. from the UAE noted similar postgraduate aspirations among dental students [29]. Similarly, Yan et al. reported that Japanese and Chinese dental students prioritized secure public-sector roles and postgraduate education [30]. Scarbecz and Ross highlighted gender differences in postgraduate aspirations among U.S. students, which aligns with our findings [31].

Single-institution studies conducted in 2011 reported that prosthodontics followed by orthodontics was the most preferred specialty among male dental students [27], whereas orthodontics followed by endodontics was the most preferred among female dental students [28, 32]. Another study conducted at the same institution in 2014 reported that oral and maxillofacial surgery followed by orthodontics was the most preferred specialty among male participants, and operative dentistry followed by pediatric dentistry was the most preferred discipline among female respondents [23]. According to Halawany et al., restorative and esthetic dentistry was the most preferred postgraduate specialty among male as well as female dental students, followed by endodontics among male and oral and maxillofacial surgery among female students [10]. The findings by Al-Hallak et al. revealed that orthodontics followed by oral surgery were the most favorable specialties for the participating students [14]. In this study, it was found that restorative and esthetic dentistry followed by oral and maxillofacial surgery and orthodontics was the specialty of choice among female students, whereas the male participants preferred oral and maxillofacial surgery followed by restorative and esthetic dentistry and endodontics.

Specialization in dentistry is rewarding, and it has been reported that dental specialists earn higher incomes compared to general dentists [29, 31]. However, general dentists meet the treatment needs of a large percentage of the population in Saudi Arabia [10]. Therefore, the economic and professional security by the government is much needed to accommodate the general dentists. Furthermore, the respondents showed less interest in periodontics, which is in line with the study done by Fidele et al. [33] Periodontal disease is one of the most common inflammatory diseases in adults worldwide that needs immediate attention from the government and the dental profession officials [34]. In Saudi Arabia, and according to one of the largest epidemiological trials in the Middle East, a multicenter clinical trial to evaluate periodontal disease in Saudi Arabia has noted that 45.0% of the general population between the ages of 18 and 59 suffer from gingival bleeding [35]. Therefore, it is recommended that the higher authorities in charge of dental postgraduate studies should encourage UGs to pursue specialization in periodontology. Other than this, none of the respondents wished to pursue postgraduation in dental public health, forensic dentistry, or oral biology. This can lead to a lack of skills and expertise in dentistry in Saudi Arabia, which will in turn impact the dental services provided to the Saudi public in the future. The lack of interest in certain specialties, such as periodontology and dental public health, echoes global trends and calls for curricular and policy interventions.

After graduation, the avenues preferred by the dental graduates may be joining private clinics, public health services, or pursuing postgraduate courses to gain expertise in a particular field [32]. The study done on the Saudi dental students noted that the majority of the participants preferred to work in the public sector rather than the private sector [10]. On the other hand, the study conducted among Iranian dental students showed that most of the respondents preferred to work in the private rather than the public sector [36]. For career option preferences, it was found that “civilian dentist in the public sector” was preferred by the majority of studied participants. This choice was the same for both the male and female participants. Similar results were found in a study conducted by Halawany et al., who concluded that the two most preferred careers were “civilian dentist in the public sector” [10]. However, according to Baharvand et al., female participants were found to be more interested than males in working in public dental careers [37]. To be able to work in the public services, as in university hospitals and other specialized dental hospitals, might have given the impression that the job is secured for a lifetime, in addition to a fixed retirement income, and this ideology is what many nations have followed for decades [30].

Academic services were selected as the second most preferred career option among the female participants and the third most preferred career option among the male participants. In Saudi Arabia, students may not be able to succeed in an academic career unless they concentrate on finishing their advanced education programs, as the majority of institutions choose teachers who are highly trained professionals in the subject. Though not statistically significant, a greater number of female than male dental students selected “researcher” in their choices. This might be attributed to the fact that an increased demand for research opportunities has a significant role to play in career promotions and for the recognition they will receive for their contribution to dentistry.

This study was undertaken as a concern for the future course of the final-year dental students in Saudi Arabia. The choice of whether to enter the workforce right away or pursue another career path, like postgraduation, is one of the hardest decisions that dental students must make. This study is able to give a better knowledge of the variables impacting students' decision to pursue advanced education, their career choices, the qualities of their future jobs, and the general perception of the need for postgraduate educational programs in Saudi Arabia's northern region. Furthermore, it can be stated that this study complements the earlier similar studies [10, 14] conducted among Saudi students and could possibly update and add more to our knowledge and understanding of the reasons for studying dentistry in Saudi Arabia.

## 5. Limitations

Certain limitations of this study should be noted when interpreting the results. As with any questionnaire-based survey, some elements of under-reporting bias might occur in the study. The cross-sectional design, nonrespondent bias and the desire of the respondents to choose socially acceptable responses, as observed in questionnaire surveys [38] may also be considered limitations. Furthermore, the present study did not permit to find if the career allied objectives would vary with time. The possibility to find factors that might influence career aspirations might have been missed. It is important to recognize that when we ask students about their future goals, we may not be able to get a true picture of what they actually do once they graduate. A follow-up study of these new aspirants within a few years of graduation could therefore help to better understand the different factors that affect their immediate educational and career goals.

Also, the small sample size of the pilot test may limit the precision of the reliability estimates, and therefore, these results should be interpreted with caution.

In addition, the study was conducted at only one dental institute in Saudi Arabia; therefore, the findings of this study should not reflect the career choices at other higher education institutions in the kingdom. However, it is worthy to note that for external validity, the results of this study were compared with the findings of other similar studies. We therefore recommend conducting multicenter studies that can provide data on the dental specialization and career options of the graduates of different colleges in Saudi Arabia.

## 6. Conclusion

Within the limitations of the study, the following conclusions were drawn:

1. The reasons for choosing dentistry as a career among the surveyed dental students were mostly related to personal and vocational factors.
2. The most preferred specialty among female respondents was restorative and esthetic dentistry, and among male respondents, it was oral and maxillofacial surgery.
3. The most preferred career among male and female respondents was to work in the public sector.

The research uncovered the key insights with strategic importance. As it is clear from the findings of this study that in the near future, a high demand for postgraduate dental education in Saudi Arabia is expected. Therefore, the results from this study will be significant to promote mentoring activities, provide guidance, and encouragement to the graduate students in selecting the most appropriate specialty and career within their capability domain. In addition, these findings can help in improving the career counseling services provided by the dental institutes of Saudi Arabia. Thus, this study can be a baseline for establishing national policies and for the improvement of graduate programs and at the same time also provide for the needs of the population [25].

## Figures and Tables

**Table 1:** Characteristics of study participants (*n* = 144).

Variable	*N* = 144	Percentage
Gender		
Male	84	58.3
Female	60	41.7
Age (years)		
Range	20–28	
Mean ± SD	23.2 ± 2.0	
Academic year		
3^rd^ year	47	32.6
4^th^ year	25	17.4
5^th^ year	47	32.6
Intern	25	17.4

**Table 2:** Differences between male and female students in their reasons for choosing dentistry as a career.

Reasons affecting choice of dentistry	Gender	Strongly agree (*n* = 5) *N* (%)	Agree (*n* = 4) *N* (%)	Neutral (*n* = 3) *N* (%)	Disagree (*n* = 2) *N* (%)	Strongly disagree (*n* = 1) *N* (%)	Median	*p*-value (Mann–Whitney test)	Effect size (*r*)
*Financial reasons*									
Easy to find jobs	Males	5 (41.7)	13 (43.3)	24 (44.4)	27 (81.8)	15 (100)	4.0	< 0.0001^∗^	0.48 (large effect)
	Females	7 (58.3)	17 (56.7)	30 (55.6)	6 (18.2)	00 (0.0)	3.0		
	Total	12 (100)	30 (100)	54 (100)	33 (100)	15 (100)	3.0	—	

Good payment	Males	15 (71.4)	24 (54.5)	27 (49.1)	15 (71.4)	03 (100)	3.0	0.919	0.01 (negligible effect)
	Females	06 (28.6)	20 (45.5)	28 (50.9)	06 (28.6)	00 (0.0)	3.0		
	Total	21 (100)	44 (100)	55 (100)	21 (100)	03 (100)	3.0	—	

*Professional reasons*									
I can be my boss	Males	17 (51.5)	26 (47.3)	33 (80.5)	08 (53.3)	00 (0.0)	3.0	0.042^∗^	0.22 (small effect)
	Females	16 (48.5)	29 (52.7)	08 (19.5)	07 (46.7)	00 (0.0)	2.0		
	Total	33 (100)	55 (100)	41 (100)	15 (100)	00 (0.0)	2.0	—	

I can practice dentistry independently after my graduation with no need to be a specialist	Males	13 (43.3)	33 (53.2)	22 (71.0)	08 (61.5)	08 (100)	2.0	0.004^∗^	0.31 (medium effect)
	Females	17 (56.7)	29 (46.8)	09 (29.0)	05 (38.5)	00 (0.0)	2.0		
	Total	30 (100)	62 (100)	31 (100)	13 (100)	08 (100)	2.0	—	

Dentistry offers more regular work hours that other medical courses	Males	09 (36.0)	35 (53.8)	29 (76.3)	07 (58.3)	04 (100)	2.0	0.001^∗^	0.34 (medium effect)
	Females	16 (64.0)	30 (46.2)	09 (23.7)	05 (41.7)	00 (0.0)	2.0		
	Total	25 (100)	65 (100)	38 (100)	12 (100)	04 (100)	2.0	—	

*Vocational reasons*									
I like to improve the esthetics of people	Males	32 (60.4)	35 (56.5)	16 (64.0)	01 (33.3)	00 (0.0)	2.0	0.732	0.04 (negligible effect)
	Females	21 (39.6)	27 (43.5)	09 (36.0)	02 (66.7)	01 (100)	2.0		
	Total	53 (100)	62 (100)	25 (100)	03 (100)	01 (100)	2.0	—	

I have good manual dexterity and skills to practice dentistry	Males	28 (53.8)	32 (58.2)	21 (67.7)	03 (60.0)	00 (0.0)	2.0	0.348	0.09 (negligible effect)
	Females	24 (46.2)	23 (41.8)	10 (32.3)	02 (40.0)	01 (100)	1.0		
	Total	52 (100)	55 (100)	31 (100)	05 (100)	01 (100)	2.0	—	

*Social reasons*									
My friend who is a dentist encouraged me to join the dental course	Males	07 (50.0)	14 (42.4)	22 (57.9)	17 (58.6)	24 (80.0)	5.0	0.006^∗^	0.30 (medium effect)
	Females	07 (50.0)	19 (57.6)	16 (42.1)	12 (41.4)	6 (20.0)	2.0		
	Total	14 (100)	33 (100)	38 (100)	29 (100)	30 (100)	3.0	—	

My family wished me to become a dentist	Males	13 (54.2)	29 (63.0)	25 (65.8)	05 (26.3)	12 (70.6)	2.0	0.705	0.05 (negligible effect)
	Females	11 (45.8)	17 (37.0)	13 (34.2)	14 (73.7)	05 (29.4)	2.0		
	Total	24 (100)	46 (100)	38 (100)	19 (100)	17 (100)	2.0	—	

Dentistry is a prestigious profession	Males	16 (64.0)	35 (66.0)	27 (60.0)	05 (31.2)	01 (20.0)	2.0	0.023^∗^	0.29 (medium effect)
	Females	09 (36.0)	18 (34.0)	18 (40.0)	11 (68.8)	04 (80.0)	2.0		
	Total	25 (100)	53 (100)	45 (100)	16 (100)	05 (100)	2.0	—	

*Personal reasons*									
My school GPA qualified me to choose dentistry as a career	Males	20 (46.5)	27 (58.7)	16 (59.3)	06 (46.2)	15 (100)	2.0	0.014^∗^	0.25 (medium effect)
	Females	23 (53.5)	19 (41.3)	11 (40.7)	07 (53.8)	00 (0.0)	2.0		
	Total	43 (100)	46 (100)	27 (100)	13 (100)	15 (100)	2.0	—	

I would get enough time for my personal life	Males	11 (37.9)	21 (52.5)	35 (71.4)	12 (60.0)	05 (83.3)	3.0	0.006^∗^	0.30 (medium effect)
	Females	18 (62.1)	19 (47.5)	14 (28.6)	08 (40.0)	01 (16.7)	2.0		
	Total	29 (100)	40 (100)	49 (100)	20 (100)	06 (100)	3.0	—	

*Note:* Effect size *r* = *Z*/$\sqrt{N}$ (if *N* = total number of observations from both groups); Interpretation: small (0.1), medium (0.3), large (0.5).

^∗^Statistically significant.

**Table 3:** Gender-wise comparison among students regarding immediate career plans upon graduation.

Future options	Gender	1^st^ choice *N* (%)	2^nd^ choice *N* (%)	3^rd^ choice *N* (%)	Maybe/not sure *N* (%)	Never *N* (%)	*p*-value (chi-square test)	Effect size (Cramer's V)
*Career option*s								
Civilian dentist in the public sector	Males	42 (56.0)	20 (26.7)	8 (10.7)	5 (6.6)	00 (0.0)	0.134	0.19 (small effect)
	Females	41 (73.2)	9 (16.1)	1 (1.8)	5 (8.9)	00 (0.0)		
	Total	83 (63.4)	29 (22.1)	9 (6.9)	10 (7.6)	00 (0.0)	—	

Academic services dentist	Males	10 (15.9)	25 (39.7)	20 (31.7)	5 (7.9)	3 (4.8)	0.292	0.14 (small effect)
	Females	6 (15.8)	14 (36.9)	7 (18.4)	7 (18.4)	4 (10.5)		
	Total	16 (15.8)	39 (38.7)	27 (26.7)	12 (11.9)	7 (6.9)	—	

Civilian dentist in the private sector	Males	3 (9.1)	10 (30.4)	11 (33.3)	8 (24.2)	1 (3.0)	0.386	0.12 (small effect)
	Females	1 (2.4)	17 (40.5)	17 (40.5)	7 (16.6)	00 (0.0)		
	Total	4 (5.4)	27 (36.0)	28 (37.3)	15 (20.0)	1 (1.3)	—	

Dentist in the military sector	Males	19 (31.7)	18 (30.0)	13 (21.6)	4 (6.7)	6 (10.0)	0.221	0.18 (small effect)
	Females	2 (9.1)	6 (27.3)	8 (36.4)	3 (13.6)	3 (13.6)		
	Total	21 (25.6)	24 (29.3)	21 (25.6)	7 (8.5)	9 (11.0)	—	

Management of dental business	Males	3 (6.4)	4 (8.5)	7 (14.9)	18 (38.3)	15 (31.9)	0.384	0.16 (small effect)
	Females	4 (19.0)	2 (9.5)	5 (23.8)	5 (23.8)	5 (23.8)		
	Total	7 (10.3)	6 (8.8)	12 (17.6)	23 (33.8)	20 (29.4)	—	

Researcher	Males	3 (10.7)	1 (3.6)	7 (25.0)	7 (25.0)	10 (35.7)	0.03^∗^	0.27 (medium effect)
	Females	4 (30.7)	3 (23.1)	00 (0.0)	1 (7.7)	5 (38.5)		
	Total	7 (17.1)	4 (9.7)	7 (17.1)	8 (19.5)	15 (36.6)	—	

Businesses outside the dental field	Males	2 (6.9)	4 (13.8)	8 (27.6)	9 (31.0)	6 (20.7)	0.154	0.15 (small effect)
	Females	0 (0.0)	5 (45.5)	4 (36.3)	1 (9.1)	1 (9.1)		
	Total	2 (5.0)	9 (22.5)	12 (30.0)	10 (25.0)	7 (17.5)	—	

Other careers	Males	2 (2.3)	2 (2.3)	10 (11.8)	28 (33.0)	43 (50.6)	0.673	0.08 (negligible effect)
	Females	2 (2.1)	4 (4.1)	18 (18.6)	31 (32.0)	42 (43.2)		
	Total	4 (2.2)	6 (3.3)	28 (15.4)	59 (32.4)	85 (46.7)	—	

*Note:* Effect size V is interpreted as small (< 0.2), medium (0.2–0.5), and large (> 0.5).

^∗^Shows that the *p* value is significant.

## Data Availability

The data set used in the current study will be made available on request from the corresponding author.
